# Mechanically gated currents in mouse sensory neurons lacking PIEZO2

**DOI:** 10.1016/j.bpj.2025.10.042

**Published:** 2025-11-01

**Authors:** Oscar Sánchez-Carranza, Valérie Bégay, Sampurna Chakrabarti, Mireia Pampols-Perez, Lin Wang, Jonathan Alexis García-Contreras, Annette Hammes, Gary R. Lewin

**Affiliations:** 1 Max Delbrück Center for Molecular Medicine in the Helmholtz Association (MDC), Molecular Physiology of Somatic Sensation Laboratory, 13125 Berlin, Germany; 2 Max Delbrück Center for Molecular Medicine in the Helmholtz Association (MDC), Molecular Pathways in Cortical Development, 13125 Berlin, Germany; 3 Charité-Universitätsmedizin Berlin, Berlin, Germany; 4 German Center for Mental Health (DZPG), Partner Site Berlin, Berlin, Germany

## Abstract

Touch sensation starts with the opening of mechanically gated activated ion channels at neuroglial endings of mechanoreceptors in the skin. The function of around half of low-threshold mechanoreceptors is dependent on the presence of the mechanically gated ion channel PIEZO2. It has been reported that particularly rapidly adapting mechanosensitive currents (RA currents) in the cell bodies of acutely cultured sensory neurons are dependent on PIEZO2. Here we re-examined this question by making a quantitative study of mechanically gated currents activated by substrate deflection in sensory neurons lacking PIEZO2. We characterized mechanically gated currents from embryonic and postnatal sensory neurons, taken from global *Piezo2*^−/−^ or *Piezo2* conditional knockouts (*Piezo2*^*cKO*^), respectively. Surprisingly, in both models, *Piezo2* gene deletion was not associated with any significant reduction in the sensitivity or incidence of mechanosensitive currents compared with wild-type controls. There was, however, a moderate reduction in the incidence of RA currents with very fast activation and inactivation kinetics in both embryonic *Piezo2*^−/−^ and juvenile *Piezo2*^*cKO*^ mice. These results show that PIEZO2 channels are not the only mechanosensitive channels mediating RA currents in sensory neurons. Furthermore, our data suggest that the phenotypes associated with *Piezo2* loss of function alleles may sometimes be due to secondary effects of gene deletion, for example, by changing the developmental trajectory of sensory neurons. Emphasis should be put on the diversity of mechanosensitive ion channel function in sensory neurons, which needs to be further elucidated.

## Significance

The mechanosensitive ion channel PIEZO2 has been proposed to be the major mechanically gated ion channel for the transduction of touch. We used patch-clamp electrophysiology to measure mechanically gated currents in sensory neurons lacking PIEZO2 channels using two genetic strategies. *Piezo2* gene deletion was associated with a moderate reduction in the frequency of very rapidly inactivating mechanosensitive currents in early postnatal sensory neurons. No significant reduction in the sensitivity or incidence of mechanosensitive currents was observed. These data challenge the idea that PIEZO2 channels are the main transducers of force in sensory neurons, at least via substrate deflection. Other interpretations should be considered, including the possibility that early loss of PIEZO2 could alter sensory neuron development to impair mechanoreceptor function.

## Introduction

The mechanically gated ion channel PIEZO2 has been linked to the senses of touch, proprioception, and pain in mammals (1,2,3,4,5,6,7,8). Touch sensation starts with the transduction of mechanical stimuli at the peripheral endings of mechanoreceptors in the skin. It has been shown that for many, but not all mechanoreceptors, the presence of PIEZO2 at sensory endings is absolutely necessary for transduction (1,2,9). However, direct measurement of mechanically gated ion channel activity at sensory endings has so far not been feasible in mammalian preparations (10), but cultured dorsal root ganglion neurons possess mechanically gated currents postulated to be functionally equivalent to those that mediate transduction at sensory endings (11,12,13,14,15). Indeed, there are several examples where loss of mechanically gated currents after genetic ablation of transduction candidates is associated with of loss of mechanoreceptor function (1,2,15,16,17). Heterologous expression of PIEZO2 channels in various cells types is associated with mechanically gated currents with very fast activation and inactivation time constants, which have been termed rapidly adapting or RA currents (8,13,14,18,19). Interestingly, RA currents measured in cells expressing PIEZO2 have kinetics that are very similar to endogenous mechanosensitive currents in isolated sensory neurons (7,13,14). However, even in the case of *Piezo2* genetic deletion in mice where the loss of RA mechanosensitive currents was described to be particularly profound, many cells still displayed mechanosensitive currents with fast kinetics (1,2).

Several years ago, we developed a technique where mechanosensitive currents can be evoked by moving the cell substrate with single pillar deflections (19,20). This method allows the quantification of current amplitude in relation to deflection amplitude and reveals mechanically gated currents with kinetics and biophysical properties almost identical to those produced by cell indentation (7,17,19,21,22). The mechanically gated ion channel ELKIN1/TMEM87a was recently identified as being necessary for normal touch receptor function in mice (17,23). Furthermore, genetic deletion of the *Elkin1* gene was associated with a loss of mechanosensitive currents in cultured sensory neurons evoked with indentation stimuli as well as pili deflection (17). Due to the postnatal lethality of mouse *Piezo2* gene ablation (24,25), there have been only limited studies on the mechanosensitive currents remaining in sensory neurons lacking PIEZO2 (1,2,26). In this study, we set out to measure mechanosensitive currents that remain in sensory neurons that genetically lack *Piezo2* in two different mouse models. Our results revealed that complete ablation of the *Piezo2* gene led to very moderate reductions in the incidence of mechanosensitive RA currents in sensory neurons. Our data strongly suggest that genetic ablation of *Piezo2* can be substantially compensated for by the presence of other mechanically gated channels in sensory neurons.

## Materials and methods

### Primary cell culture

DRG neurons were collected from all spinal segments (or only lumbar if indicated) in plating medium on ice (DMEM-F12 (Invitrogen) supplemented with L-glutamine (2 μM, Sigma-Aldrich), glucose (8 mg/mL, Sigma-Aldrich), penicillin (200 U/mL)-streptomycin (200 μg/mL), and 10% fetal horse serum). DRGs were treated with Collagenase IV (1 mg/mL, Sigma-Aldrich) for 15 min for embryonic cells (E18.5) and cells from pups (P6), at 37°C and then washed three times with Ca^2+^- and Mg^2+^-free PBS. Samples were then incubated with 0.05% trypsin (Invitrogen, Karlsruhe) at 37°C for 15 min. Collected tissue was triturated with a pipette tip and plated in a droplet of plating medium on the silanized elastomeric pillar arrays precoated with laminin (4 μg/cm^2^, Invitrogen) as described (see elastomeric pillar arrays section) (19,20). Cells were cultured overnight, and electrophysiology experiments were preformed 18–24 h after dissection.

### Mice

*Piezo2*^*+/−*^ mice (1,25) were mated to generate *Piezo2*^−/−^ mice (*Piezo2*^*KO*^). The females were monitored for 7 days to observe the presence or absence of vaginal plug during mating. The day when the vaginal plug was observed was considered as embryonic day 0.5 (E0.5). Females were sacrificed at E18.5 in a 100% CO_2_ chamber. The embryos were collected and put in PBS on ice before DRG extraction, as previously described (27). A piece of the tail was cut for genotyping.

*Hoxb8*^*+/Cre*^ (28) mice (Tg(Hoxb8-cre)^1403Uze^; MGI:4881836) were mated with *Piezo2*^*+/−*^ to generate *Hoxb8*^*+/Cre*^*;Piezo2*^*+/−*^ animals. In parallel, *Piezo2*^*fl/fl*^ mice (24) were mated with *Ai14*^*f/f*^ mice (29) (Gt(ROSA)^26Sortm14(CAG-tdTomato)Hze^; MGI:3809524) to generate *Piezo2*^*fl/fl*^*;Ai14*^*fl/fl*^. Subsequently, *Hoxb8*^*+/Cre*^*;Piezo2*^*+/−*^ were mated with *Piezo2*^*fl/fl*^*;Ai14*^*fl/fl*^ animals to generate *Piezo2*^*cKO*^ (*Hoxb8*^*+/Cre*^*; Piezo2*^*-/fl*^*;Ai14*^*+/f*^). *Hoxb8*^*+/+*^*;Piezo2*^*-/f*^*;Ai14*^*+/f*^ were used as control animals (*Piezo2*^*Ctrl*^). Littermates were used in each experiment. All experiments with mice were done in accordance with protocols reviewed and approved by the German Federal authorities (State of Berlin).

### Genotyping

To genotype *Piezo2*^*K*O^ and *Piezo2*^*cKO*^ animals, a piece of tail was taken from E18.5 embryos and P1 tattooed pups, respectively. Similar to previous studies, we found that *Piezo2*^*ko*^ embryos were found at ratios consistent with Mendelian inheritance (25). Tissues were incubated overnight at 55°C while shaking at 800 rpm in a lysis buffer containing (in mM) 200 NaCl, 100 Tris (pH 8.5), 5 EDTA, 0.2% of SDS, and Proteinase K (10 mg/mL, Carl Roth). PCRs were performed using supernatant of the lysis preparation as DNA template (20–100 ng), 1× Taq PRC buffer, 2 mM MgCl_2_, 0.2 mM dNTPs, 1.25U Taq-polymerase (Thermo-Fisher Scientific), and 5 pmol of forward- and reverse-specific primers targeting *piezo2* (Table 1).

**Table 1:** PCR primers for animal genotyping

Mouse line	Forward primer	Reverse primer	Expected size (bp)
*Piezo2*^*KO*^ for WT allele	CTC AGA CTT GGA GAT CCT GTA GC	CCC TAC CCA CCC ATT CCC ATT TT	140
*Piezo2*^*KO*^ for mutant allele	CTC AGA CTT GGA GAT CCT GTA GC	CTT CCT GAC TAG GGG AGG AGT A	392
*Hoxb8*^*Cre*^	GGG GTC TCT AAT GGA TGC AA	AAC CAG CGT TTT CGT TCT GC	1200
*Piezo2*^*fl*^	CTC AGA CTT GGA GAT CCT GTA GC	GAC TCA GAT TTT CCA CAT GGG G	258
*Ai14* for WT allele	AAG GGA GCT GCA GTG GAG TA	CCG AAA ATC TGT GGG AAG TC	297
*Ai14* for mutant allele	CTG TTC CTG TAC GGC ATG G	GGC ATT AAA GCA GCG TAT CC	196

### Elastomeric pillar arrays

Elastomeric pillar arrays were prepared as previously described (19). Briefly, freshly mixed PDMS (Sylgard 184, Dow Corning, USA) was prepared in a 1:10 mix of both components and incubated for 30 min in vacuum. Negative masters were covered with PDMS and left for 30 min. Borosilicate glass coverslips (22 × 22 mm, VWR International, Thickness No. 2) were incubated for 2 min in a FEMTO plasma cleaner (Diener Electronic, Nagold, Germany) for oxygen plasma treatment activation, and the activated side (upper part) was placed (side down) on the PDMS-covered negative masters. Pillar arrays were incubated at 100°C for 1 h. Pillar arrays were pealed apart from the negative masters and were activated by oxygen plasma treatment for 2 min. Each pilus within the array exhibited a radius of 1.79 μm and a length of 5.8 μm. Pillar arrays were silanized using vapor phase (tridecafluoro-1,1,2,2-tetrahydrooctyl) trichlorosilane 97% (AB111444, ABCR GmbH & Co. KG, Karlsruhe, Germany) for 45 min and then coated with laminin (4 μg/cm^2^, Invitrogen) overnight at 37°C in a humid chamber.

### Electrophysiology

Patch-clamp experiments were performed at room temperature. Whole-cell patch-clamp experiments were carried out in sensory neurons using pulled and heat-polished borosilicate glass pipettes (Harvard apparatus, 1.17 mm × 0.87 mm) with a resistance of 3–6 MΩ. The pipettes were pulled using a DMZ puller (Germany) and filled with a solution containing (in mM) 110 KCl, 10 NaCl, 1 MgCl_2_, 1 EGTA, and 10 HEPES. The pH was adjusted to 7.3 with KOH. QX-314 (Alomone Labs) at 1 μM and was added to the intracellular solution. The extracellular solution contained (in mM) 140 NaCl, 4 KCl, 2 CaCl_2_, 1 MgCl_2_, 4 glucose, and 10 HEPES. The pH was adjusted to 7.4 with NaOH. Pipette and membrane capacitance were compensated using the auto-function of Patchmaster (HEKA, Elektronik, Germany) and series resistance was compensated to minimize voltage errors. Currents were evoked by mechanical stimuli (see below) at a holding potential of −60 mV. DRG recordings from *Piezo2*^*+/+*^, *Piezo2*^*+/−*^, and *Piezo2*^−/−^ embryos were carried out in a blind manner. When recording neurons from *Piezo2*^*cKO*^, only red cells (td-Tomato+ cells) were selected.

For pillar arrays experiments, a single pilus was deflected using a heat-polished borosilicate glass pipette (mechanical stimulator) driven by a MM3A micromanipulator (Kleindiek Nanotechnik, Germany) as described in (19). Pillar deflection stimuli were applied in the range of 1–1000 nm, and larger deflections were discarded. Data were analyzed using Fitmaster software (HEKA Electronik, Germany). Mechanically gated currents were classified according to their inactivation kinetics as previously described: the rapidly adapting (RA, τ_inact_ < 5 ms), intermediate adapting (IA, τ_inact_ 5–50 ms), and slowly adapting currents (SA, τ_inact_ > 50ms) (19). For quantification and comparison analysis, data were binned by the magnitude of the stimuli (1–10, 11–50, 51–100, 101–250, 251–500, 501–1000 nm) and calculated the mean of the current amplitudes within each bin for every cell. Bright-field images (Zeiss 200 inverted microscope) were collected using a 40× objective and a CoolSnapEZ camera (Photometrics, Tucson, AZ) before and during the pillar stimuli to calculate the pillar deflection. The pillar movement was calculated comparing the light intensity of the center of each pilus before and after the stimuli with a 2D Gaussian fit (Igor Software, WaveMetrics, USA).

### Single-molecule fluorescence in situ hybridization (smFISH)

Lumbar DRGs were collected from P6 pups and were incubated for 40 min in 4% para-formaldehyde washed with PBS and incubated in 30% sucrose (in PBS) overnight at 4°C. DRGs were embedded in OCT Tissue Tek (Sakura, Alphen aan den Rijn). Cryosections of thickness of 10 μm were stored at −80°C until used for experiments. In situ hybridization was carried out according to the manufacturer’s instructions (RNAscopeTM Multiplex Fluorescent V2 assay, ADC, Kit #323110, *piezo2* mouse probe #439971). LSM700 Carl Zeiss and CSU-WI Olympus spinning disk confocal microscopes were used to acquired images at 20× and numerical aperture 0.5 and 0.8, respectively. Fluorescence intensity was analyzed using Fiji21.

### Statistical analysis

All data analyses were performed using GraphPad Prism, and all data sets were tested for normality. Parametric data sets were compared using a two-tailed, Student's *t*-test. Nonparametric data sets were compared using a Mann-Whitney test. To compare more than two groups, one-way ANOVA was used. Categorical data were compared using Fisher’s exact or χ2 tests. Power analyses indicated that the sample sizes were large enough to detect a reduction of 70% in the incidence of MA currents as previously published (1,2).

## Results and discussion

**Figure 1:**
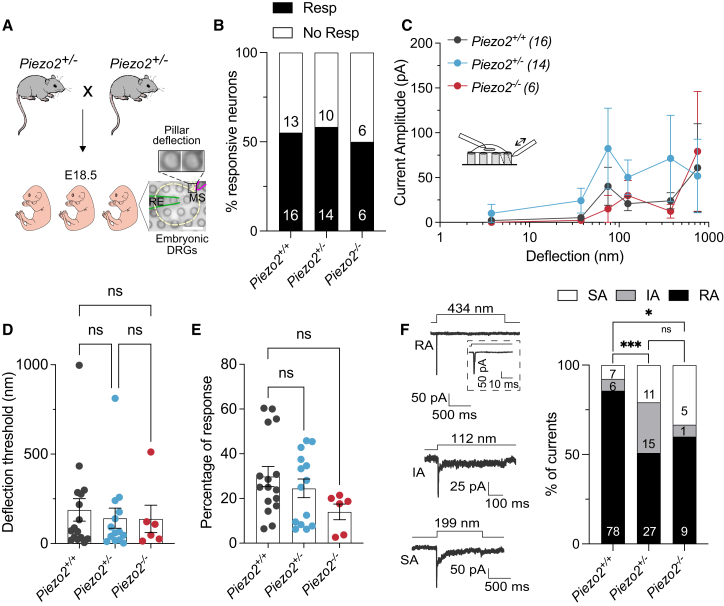
Embryonic DRG neurons from *Piezo2*^−/−^ displayed deflection-gated currents. (*A*) Cartoon representing the acquisition of embryonic DRG neurons at E18.5. (*Right*) Bright-field image of an embryonic DRG neuron cultured on pillar arrays. In the insert, the position of a single pilus is shown before and during the deflection stimulus. (*B*) Stacked histogram showing the percentage of responsive (Resp) and nonresponsive (No Resp) cells to pillar deflection. Numbers indicate the number of cells. (*C*) Stimulus-response plot of the deflection sensitive currents from embryonic DRGs in *Piezo2*^*+/+*^ (*black*), *Piezo2*^*+/−*^ (*blue*), and *Piezo2*^−/−^ (*red*) mice. Data are plotted as mean ± SEM. (*D*) No differences in deflection thresholds to activate mechanically gated currents were observed between DRGs from wild-type and mutants. Deflection threshold was calculated as the smallest deflection stimulus applied that evoked a current. Each dot represents one cell. (mean ± SEM.) (*E*) The percentage of response was statistically similar in all genotypes. Percentages were calculated according to the total amount of stimulations applied correlate with the stimuli that evoked currents (Kruskal-Wallis test; *Piezo2*^*+/+*^ vs. *Piezo2*^*+/−*^, *p* > 0.999; *Piezo2*^*+/+*^ vs. *Piezo2*^−/−^*, p* = 0.07). (*F*) (*Left*) Representative traces of the three different types of deflection currents in embryonic DRG neurons: RA, IA, and SA currents. The insert shows expanded current traces. Deflection stimuli applied are indicated for each trace. (*Right*) The percentage of RA currents decreases in DRG neurons from *Piezo2*^*+/−*^ and *Piezo2*^−/−^ mice compared with wild-type. Values in the histograms indicate the number of the currents observed (Fisher’s exact test; ^∗∗∗^*p* < 0.001, ^∗^*p* = 0.016).

By using cultured sensory neurons from *Piezo2*^−/−^ mice, we could record mechanically activated currents (MA currents) that must have a molecular composition independent of PIEZO2 channels. This experiment was challenging as sensory neurons can only be recorded at the end of embryonic development (E18.5) as *Piezo2*^−/−^ mice die shortly after birth (25). We made sensory neuron cultures from all E18.5 embryos obtained from pregnant mice derived from *Piezo2*^*+/−*^ matings and obtained the genotypes using a rapid PCR genotyping protocol (see materials and methods). Sensory neurons were plated on pillar arrays so we could use patch-clamp electrophysiology to measure deflection-evoked currents (Fig. 1 *A*). The pillar technique is a sensitive way to measure mechanically gated currents mediated by PIEZO1 and PIEZO2 channels (7,19,21,22), as well as other mechanically gated channels like ELKIN1 and TRPV4 (17,21,23). Using indentation stimuli, it was shown that the proportion of sensory neurons exhibiting MA currents increases from around 60% at E13.5 to near 80% at birth (17,27). To characterize deflection-gated currents in sensory neurons at embryonic stage E18.5, embryonic DRG neurons from the *Piezo2*^*+/*+^, *Piezo2*^*+/−*^, and *Piezo2*^−/−^ mice were dissociated and plated on elastomeric pillar arrays precoated with laminin. Twenty-four hours after plating, MA currents were recorded as previously described (7,17,19,21). In cultures from adult DRGs, we previously showed that around 70% of the cells displayed deflection-gated currents (7,17). In this study, we found that only around half of the E18.5 wild-type neurons displayed deflection-evoked currents using stimuli between 1 and 1000 nm, 16/29 neurons from 16 *Piezo2*^*+/+*^ embryos (Fig. 1 *B*). Surprisingly, the proportion of responsive cells recorded from *Piezo2*^−/−^ embryos (6/12 neurons from six embryos) was not different compared to controls or to neurons recorded from *Piezo2*^*+/−*^ embryos (14/24 neurons from 14 embryos). Additionally, the mean amplitudes of deflection-evoked currents for a range of stimuli were also not different between the three genotypes (Fig. 1 *C* and *D*). In responsive cells we observed a reduction in the percentage of deflection stimuli that evoked a current in *Piezo2*^−/−^ neurons compared with *Piezo2*^*+/+*^ neurons, yet this small difference was not statistically significant (Fig. 1 *E*). As in previous studies using pillar stimuli or indentation (7,13,15,16,19,27), sensory neurons displayed deflection-gated currents with different inactivation time constants, rapidly adapting (RA, τ_inact_ < 5 ms), intermediately adapting (IA, τ_inact_ 5–50 ms), or slowly adapting (SA, τ_inact_ > 50ms) (Fig. 1 *F*). For all three genotypes, most of the currents recorded were RA; however, these currents made up a substantially smaller proportion of the total in both *Piezo2*^−/−^ and *Piezo2*^*+/−*^ sensory neurons, which was statistically significant compared with *Piezo2*^*+/+*^ neurons (Fig. 1 *F*). Consistent with this analysis the mean inactivation kinetics (τ_inact_) of all mechanically gated currents recorded from *Piezo2*^*+/−*^ and *Piezo2*^−/−^ neurons were 2.5 to fivefold slower compared with those recorded from *Piezo2*^*+/+*^neurons (Table 2). The speed of current activation (τ_act_) was also significantly slowed in neurons from *Piezo2*^*+/−*^ and *Piezo2*^−/−^ embryos, as were the latencies for current activation compared with those measured in *Piezo2*^*+/+*^neurons (Table 2).

**Table 2:** Electrophysiological properties of currents recorded from embryonic DRG neurons

	Piezo2^+/+^	Piezo2^*+/−*^	Piezo2^−/−^
Cells (no. of currents)	16 (91)	14 (53)	6 (15)
Latency (ms)	2.05 ± 0.19	2.32 ± 0.42	5.31 ± 0.8^∗∗∗^
*t*_act_ (ms)	1.22 ± 0.14	2.18 ± 0.4^∗^	4.16 ± 1.1^∗∗^
*t*_inact_ (ms)	18.65 ± 9.7	54.96 ± 15.48^∗∗∗^	97.4 ± 42.74^∗∗^

*^∗^p = 0.011, ^∗∗^p < 0.01, ^∗∗∗^p < 0.001* (Mann-Whitney test) means ± SEM.

**Figure 2:**
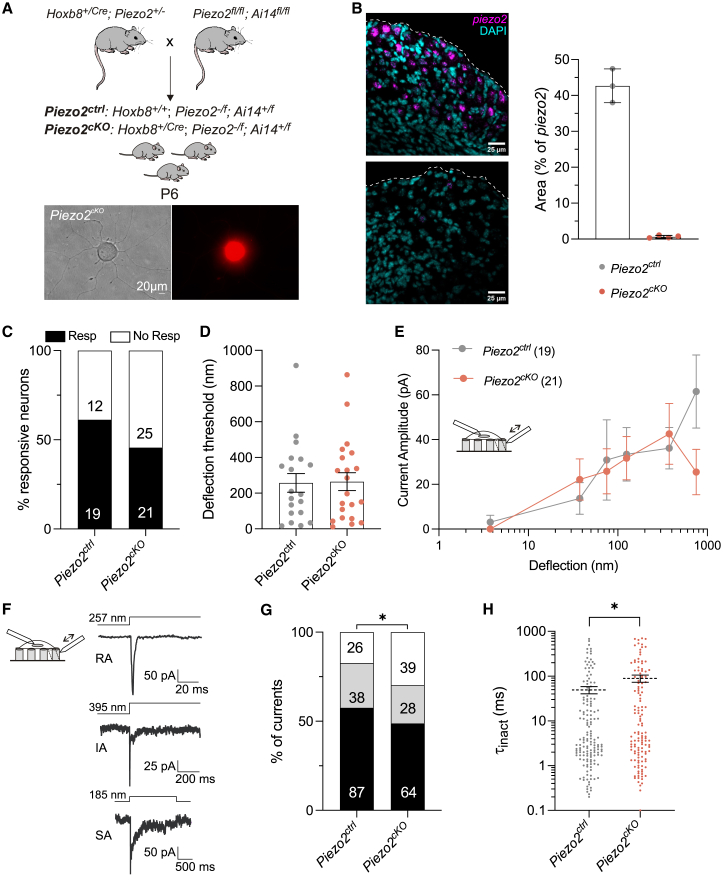
Deflection-gated currents evoked in *Piezo2*^cKO^ neurons. (*A*) (*Above*) Scheme for the generation of *Piezo2*^*ctrl*^ and *Piezo2*^*cKO*^ animals. (*Below*) Acutely prepared dorsal DRG neurons from *Piezo2*^*cKO*^ expressing td-Tomato (*red cells*). (*B*) (*Left*) Representative pictures of *piezo2* in situ hybridization in sections from P6 lumbar dorsal root ganglia. Magenta, *piezo2* mRNA; cyan, 4′,6-diamidino-2-phenylidole (DAPI). Dashed lines show outline of the section. Scalebar, 25 μm. (*Right*) Quantification of area of *piezo2* transcript signal. Each dot represents mean values from sections from one mouse mean ± SEM. (*C*) Stacked histogram showing the percentage of responsive and nonresponsive (No Resp) dorsal DRG neurons to pillar deflection. Numbers are cells (*p* > 0.05, χ^2^ test). (*D*) The deflection threshold in neurons from *Piezo2*^*cKO*^ mice was similar to controls. (*E*) Stimulus-response plot of the deflection sensitive currents in sensory neurons from *Piezo2*^*ctrl*^ (*gray*) and *Piezo2*^*cKO*^ mice (*salmon*). The large circles are plotted as mean ± SEM. (*F*) (*Left*) Representative traces of the three different types of mechanically gated currents in sensory neurons from P6 animals. RA, IA, and SA currents were observed. The deflection stimuli applied are indicated for each trace. (*G*) The percentage of RA currents was slightly reduced in neurons from *Piezo2*^*cKO*^ mice. The numbers in the histograms represent the number of the currents observed (*p* = 0.04; χ^2^ test). (*H*) Plot showing that τ_inact_ kinetics are slower in *Piezo2*^*cKO*^ compared with controls mouse (Mann-Whitney test, ^∗^*p* = 0.042).

We were surprised to see no substantial loss of mechanically gated currents in embryonic sensory neurons, as it has been reported that conditional *Piezo2* gene deletion was associated with a substantial loss of RA currents in adult sensory neurons (1,2). We thus generated *Piezo2*^*cKO*^ mice by crossing *Hoxb8*^*+/Cre*^*;Piezo2*^*+/−*^ mice with *Piezo2*^*fl/fl*^*; Ai14*^*fl/fl*^ mice to obtain the following genotypes (*Hoxb8*^*+/Cre*^*:Piezo2*^*-/f*^*:Ai14f*^*+/f*^, termed here *Piezo2*^*cKO*^), the control pups had the following genotype (*Hoxb8*^*+/+*^*:Piezo2*^*-/f*^*:Ai14f*^*+/f.*^). In our *Piezo2*^*cKO*^ mice, there is an early and complete deletion of the *Piezo2* gene in lumbar sensory neurons, which additionally express td-Tomato (Fig. 2 *A*). As reported previously, *Piezo2*^*cKO*^ animals started to develop hindlimb movement deficits presumed to derive from loss of proprioception (4). We observed that our *Piezo2*^*cKO*^ animals already at postnatal day 6 (P6) could not right themselves from a supine position (Video S1). We verified that in the DRG of *Piezo2*^*cKO*^, virtually no *Piezo2* mRNA was detectable as previously reported (2) (Fig. 2 *B*). We made cultures of P6 sensory neurons as our regulatory authorities judged that the burden of the phenotype did not justify the study of these mice at more mature stages. With our strategy, *Piezo2*^*cKO*^ sensory neurons showed expression of td-Tomato, allowing us to unequivocally identify sensory neurons in culture devoid of PIEZO2. (Fig. 2 *A*). Around 60% (19/31 cells from six pups) of *Piezo2*^*ctrl*^ neurons showed deflection-evoked currents, but only around 45% (21/46 cells from six pups) displayed currents from *Piezo2*^*cKO*^ mice. This small reduction was, however, not statistically significant (Fishers exact test *p* = 0.25) (Fig. 2 *C*). As for embryonic sensory neurons, we did not observe differences in the deflection-current amplitude relationship between control neurons and those from *Piezo2*^*cKO*^ mutants (Fig. 2 *D* and *E*).

Video S1. Two P6 pups, one control (*left*) and one Piezo2cKO mouse (*right*), are shown being turned over onto their backs Note that the wild type mouse was quickly able to right itself, but the mutant pup was unable to right itself in the time frame of this video.

The three types of currents, RA, IA, and SA, were observed in control and mutant cells (Fig. 2 *F*). *Piezo2*^*cKO*^ neurons showed a 10% reduction of RA currents compared with control cells (Fig. 2 *G*). This again indicates that some RA currents are *Piezo2* dependent. Consistent with the reduction in the percentage of RA currents, we measured more currents in *Piezo2*^*cKO*^ cells with slower inactivation time constants compared with control neurons (Fig. 2 *G* and *H*; Table 3). Since our controls in this case are heterozygote for *Piezo2* gene deletion, it is possible that the magnitude of the reduction in RA currents was underestimated in these experiments.

**Table 3:** Biophysical properties of currents recorded from *Piezo2*^*cKO*^ DRG neurons

	Piezo2^ctrl^	Piezo2^cKO^
Cells (no. of currents)	19 (151)	21 (132)
Latency (ms)	5.68 ± 0.63	5.34 ± 0.6
*t*_act_ (ms)	1.35 ± 0.12	1.78 ± 0.16
*t*_inact_ (ms)	49.3 ± 9.1	88.84 ± 16.21^∗^

*^∗^p = 0.04* (Mann-Whitney test).

It has been widely accepted that the PIEZO2 protein is the major transducer of mechanical force in touch receptor neurons (30). Force is transduced at the cutaneous neuroglial endings of mechanoreceptors (31), and genetic ablation of PIEZO2 channels leads to silencing of around 40% of these neurons (1,2). Loss or silencing of mechanoreceptor function has been associated with loss of mechanosensitive currents in adult cultured sensory neurons (1,2,15,17). Previous studies using two *Piezo2* conditional knockout models also reported a substantial loss of RA mechanosensitive currents in adult cultured sensory neurons (1,2). Here we re-examined this issue by recording mechanosensitive currents in late embryonic sensory neurons from *Piezo2* constitutive knockouts as well as in a *Piezo2* conditional knockout model. In contrast to previous reports, we observed no substantial loss of mechanosensitive currents in both models. We did measure a moderate reduction in the incidence of the fastest RA currents, suggesting that PIEZO2 channels contribute to some of the RA currents measured in sensory neurons. It is possible that more moderate reductions in the incidence of MA currents were not detected due to the insufficient sample sizes, although these were similar to previous studies (1,2). In this study, we used the pillar method to measure substrate deflection induced currents, instead of the indentation technique used in earlier studies. However, both indentation and pillar deflection are efficient ways to activate heterologously expressed PIEZO channels (18,19,21,23), and similar mechanosensitive currents are found using both methods in sensory neurons (7,15,17,19). Indeed, recent data show that these methods applied to study the same mutant mice produce results that are broadly in agreement (7,15,17,19). Another difference between this and earlier studies is that we recorded mechanosensitive currents in late embryonic and early postnatal sensory neurons rather than in adults. It is well documented that mechanoreceptor function matures postnatally (32), and this may be influenced by dynamic changes in expression or function of mechanosensitive channels. The relative lack of effect of *Piezo2* deletion on mechanosensitive currents might suggest that PIEZO2 channels only become important in the mature somatosensory system. However, we observed robust expression of *Piezo2* at P6 (Fig. 2), and these mice already developed motor abnormalities, suggesting that proprioceptor function was already impaired.

The genetic loss of an ion channel can lead to changes in the structure and function of neurons not directly related to loss of ion channel activity. For example, loss of function mutations in the sensory voltage-gated sodium channel Na_V_1.7 are associated with morphological changes in the sensory endings of nociceptors in the skin (33). Mutations that change the biophysical function of ion channels have been used to validate whether the channel contributes to endogenous mechanosensitive channel function (34). In this context, gain of function mutations in the PIEZO2 channel introduced into the mouse were recently shown to have minimal effects on mechanoreceptor function (7) and no effect on proprioceptor function (35). In light of these findings, we should ask why do loss of function mutations lead to such profound changes in touch and proprioception in mice and humans (1,2,5). Touch is transduced at the neuroglial endings in the skin, but recent data indicate that sensory Schwann cells actively participate in the transduction of light touch (31,36). Thus, mechanosensitive channels in the sensory neuron membrane may not be the only determinants of mechanoreceptor function in vivo. Finally, recent work has shown that PIEZO proteins bind MyoD (myoblast determination) family inhibitor proteins (MDFIC and MDFI), which are transcription factor regulators (37,38). PIEZO2 sequestration of MDFIC proteins could have significant developmental consequences as pathological variants in the *MDFIC* gene cause perinatal death due to aberrant development of the lymphatic system (38). It was also recently shown with gain and loss of function experiments that PIEZO2 plays a role in heart development, by influencing the migration and assembly of endothelial cells in the coronary arteries (22). Thus, loss of PIEZO2 proteins could in principle alter developmental trajectories of different cell types, including perhaps sensory neurons. In conclusion, our study shows that PIEZO2 is just one of an as yet unknown number of channels that contribute to sensory neuron mechanosensitivity.

## Data and code availability

Data of this study are available from the corresponding author upon reasonable request.

## Acknowledgments

We thank Franziska Bartelt, Maria Braunschweig, and Kathleen Barda for technical assistance.

Support was provided by 10.13039/501100001659Deutsche Forschungsgemeinschaft (G.R.L. and A.H., SFB958-B6) and 10.13039/501100000781European Research Council grant to (G.R.L, ERC 789128). L.W. was supported by the Chinese Scholarship Council and funding from the German Center for Mental Health (DZPG), funded by the 10.13039/501100002347Federal Ministry of Education and Research. Support was provided by the Helmholtz Institute for Translational AngioCardio Science (HI-TAC) to O.S.-C., M.P.-P., and A.H and 10.13039/501100001659Deutsche Forschungsgemeinschaft (DFG GRK 2318–318905415-B1) to A.H.

## Author contributions

Conceptualization: O.S.-C., V.B., and G.R.L.; mouse model design, validation, and regulatory issues: V.B. and G.R.L.; electrophysiology: O.S.-C. with the help of S.C. and J.AG.-C.; in situ hybridization: M.P.-P., L.W., and O.S.-C.; funding acquisition: G.R.L. and A.H.; supervision: G.R.L. and A.H.; writing original draft: O.S.-C. and G.R.L.; writing review and editing: O.S.-C. and G.R.L.

## Declaration of interests

The authors declare no competing interests.

## References

[1] Ranade S.S., Woo S.-H., Patapoutian A. (2014). Piezo2 is the major transducer of mechanical forces for touch sensation in mice. Nature.

[2] Murthy S.E., Loud M.C., Patapoutian A. (2018). The mechanosensitive ion channel Piezo2 mediates sensitivity to mechanical pain in mice. Sci. Transl. Med..

[3] Szczot M., Liljencrantz J., Chesler A.T. (2018). PIEZO2 mediates injury-induced tactile pain in mice and humans. Sci. Transl. Med..

[4] Woo S.-H., Lukacs V., Patapoutian A. (2015). Piezo2 is the principal mechanotransduction channel for proprioception. Nat. Neurosci..

[5] Chesler A.T., Szczot M., Bönnemann C.G. (2016). The role of PIEZO2 in human mechanosensation. N. Engl. J. Med..

[6] Fernández-Trillo J., Florez-Paz D., Gomis A. (2020). Piezo2 Mediates Low-Threshold Mechanically Evoked Pain in the Cornea. J. Neurosci..

[7] Sánchez-Carranza O., Chakrabarti S., Lewin G.R. (2024). Piezo2 voltage-block regulates mechanical pain sensitivity. Brain.

[8] Coste B., Mathur J., Patapoutian A. (2010). Piezo1 and Piezo2 Are Essential Components of Distinct Mechanically Activated Cation Channels. Science.

[9] Hoffman B.U., Baba Y., Lumpkin E.A. (2022). Focused ultrasound excites action potentials in mammalian peripheral neurons in part through the mechanically gated ion channel PIEZO2. Proc. Natl. Acad. Sci. USA.

[10] Nikolaev Y.A., Ziolkowski L.H., Bagriantsev S.N. (2023). 3D architecture and a bicellular mechanism of touch detection in mechanosensory corpuscle. Sci. Adv..

[11] McCarter G.C., Reichling D.B., Levine J.D. (1999). Mechanical transduction by rat dorsal root ganglion neurons in vitro. Neurosci. Lett..

[12] Drew L.J., Wood J.N., Cesare P. (2002). Distinct mechanosensitive properties of capsaicin-sensitive and -insensitive sensory neurons. J. Neurosci..

[13] Hu J., Lewin G.R. (2006). Mechanosensitive currents in the neurites of cultured mouse sensory neurones. J. Physiol..

[14] Coste B., Crest M., Delmas P. (2007). Pharmacological dissection and distribution of NaN/Nav1.9, T-type Ca2+ currents, and mechanically activated cation currents in different populations of DRG neurons. J. Gen. Physiol..

[15] Wetzel C., Hu J., Lewin G.R. (2007). A stomatin-domain protein essential for touch sensation in the mouse. Nature.

[16] Wetzel C., Pifferi S., Lewin G.R. (2017). Small-molecule inhibition of STOML3 oligomerization reverses pathological mechanical hypersensitivity. Nat. Neurosci..

[17] Chakrabarti S., Klich J.D., Lewin G.R. (2024). Touch sensation requires the mechanically gated ion channel ELKIN1. Science.

[18] Ojeda-Alonso J., Bégay V., Lewin G.R. (2022). Lack of evidence for participation of TMEM150C in sensory mechanotransduction. J. Gen. Physiol..

[19] Poole K., Herget R., Lewin G.R. (2014). Tuning Piezo ion channels to detect molecular-scale movements relevant for fine touch. Nat. Commun..

[20] Shrestha S., Richardson J., Poole K., Zaidel-Bar R. (2023). Mechanobiology: Methods and Protocols.

[21] Servin-Vences M.R., Moroni M., Poole K. (2017). Direct measurement of TRPV4 and PIEZO1 activity reveals multiple mechanotransduction pathways in chondrocytes. eLife.

[22] Pampols-Perez M., Fürst C., Hammes A. (2025). Mechanosensitive PIEZO2 channels shape coronary artery development. Nat. Cardiovasc. Res..

[23] Patkunarajah A., Stear J.H., Poole K. (2020). TMEM87a/Elkin1, a component of a novel mechanoelectrical transduction pathway, modulates melanoma adhesion and migration. eLife.

[24] Woo S.H., Ranade S., Patapoutian A. (2014). Piezo2 is required for Merkel-cell mechanotransduction. Nature.

[25] Nonomura K., Woo S.-H., Patapoutian A. (2017). Piezo2 senses airway stretch and mediates lung inflation-induced apnoea. Nature.

[26] Murthy S.E. (2023). Deciphering mechanically activated ion channels at the single-channel level in dorsal root ganglion neurons. J. Gen. Physiol..

[27] Lechner S.G., Frenzel H., Lewin G.R. (2009). Developmental waves of mechanosensitivity acquisition in sensory neuron subtypes during embryonic development. EMBO J..

[28] Witschi R., Morscher G., Zeilhofer H.U. (2010). Hoxb8-Cre Mice : A Tool for Brain-Sparing Conditional Gene Deletion. Genesis.

[29] Madisen L., Zwingman T.A., Zeng H. (2010). A robust and high-throughput Cre reporting and characterization system for the whole mouse brain. Nat. Neurosci..

[30] Handler A., Ginty D.D. (2021). The mechanosensory neurons of touch and their mechanisms of activation. Nat. Rev. Neurosci..

[31] Ojeda-Alonso J., Calvo-Enrique L., Lewin G.R. (2024). Sensory Schwann cells set perceptual thresholds for touch and selectively regulate mechanical nociception. Nat. Commun..

[32] Fitzgerald M. (1987). Cutaneous primary afferent properties in the hind limb of the neonatal rat. J. Physiol..

[33] McDermott L.A., Weir G.A., Bennett D.L. (2019). Defining the Functional Role of NaV1.7 in Human Nociception. Neuron.

[34] O’Hagan R., Chalfie M., Goodman M.B. (2005). The MEC-4 DEG/ENaC channel of Caenorhabditis elegans touch receptor neurons transduces mechanical signals. Nat. Neurosci..

[35] Ma S., Dubin A.E., Patapoutian A. (2023). Excessive Mechanotransduction in Sensory Neurons Causes Joint Contractures. Science.

[36] Abdo H., Calvo-Enrique L., Ernfors P. (2019). Specialized cutaneous Schwann cells initiate pain sensation. Science.

[37] Zhou Z., Ma X., Cox C.D. (2023). MyoD-family inhibitor proteins act as auxiliary subunits of Piezo channels. Science.

[38] Byrne A.B., Brouillard P., Harvey N.L. (2022). Pathogenic variants in MDFIC cause recessive central conducting lymphatic anomaly with lymphedema. Sci. Transl. Med..

